# Prosecutor: parameter-free inference of gene function for prokaryotes using DNA microarray data, genomic context and multiple gene annotation sources

**DOI:** 10.1186/1471-2164-9-495

**Published:** 2008-10-21

**Authors:** Evert Jan Blom, Rainer Breitling, Klaas Jan Hofstede, Jos BTM Roerdink, Sacha AFT van Hijum, Oscar P Kuipers

**Affiliations:** 1 Molecular Genetics, Groningen Biomolecular Sciences and Biotechnology Institute, University of Groningen, the Netherlands; 2 Groningen Bioinformatics Centre, University of Groningen Kerklaan 30, 9751 NN, Haren, the Netherlands; 3 Institute for Mathematics and Computing Science, University of Groningen, Nijenborgh 9, 9747 AG, Groningen, the Netherlands; 4 Current address: NIZO Food Research, Kernhemseweg 2, 6718 ZB, Ede the Netherlands

## Abstract

**Background:**

Despite a plethora of functional genomic efforts, the function of many genes in sequenced genomes remains unknown. The increasing amount of microarray data for many species allows employing the guilt-by-association principle to predict function on a large scale: genes exhibiting similar expression patterns are more likely to participate in shared biological processes.

**Results:**

We developed Prosecutor, an application that enables researchers to rapidly infer gene function based on available gene expression data and functional annotations. Our parameter-free functional prediction method uses a sensitive algorithm to achieve a high association rate of linking genes with unknown function to annotated genes. Furthermore, Prosecutor utilizes additional biological information such as genomic context and known regulatory mechanisms that are specific for prokaryotes. We analyzed publicly available transcriptome data sets and used literature sources to validate putative functions suggested by Prosecutor. We supply the complete results of our analysis for 11 prokaryotic organisms on a dedicated website.

**Conclusion:**

The Prosecutor software and supplementary datasets available at  allow researchers working on any of the analyzed organisms to quickly identify the putative functions of their genes of interest. A *de novo *analysis allows new organisms to be studied.

## Background

One of the central challenges in computational biology is the prediction of gene function [1]. The inference of gene function typically starts with DNA sequence analysis based on ortholog information [2-5]. Although this method has proven to be successful in many cases, considerable numbers of genes (20–50%) in current genome annotations still are of unknown function. Complementary approaches are therefore required to characterize the function of these genes.

Since the start of the DNA microarray era, the "guilt-by-association" (GBA) methodology has been used to infer gene function [6-9]. This concept is based on the assumption that genes involved in similar cellular functions are likely to display correlated expression behavior [10-12]. In addition, this correlated behavior might identify common regulatory mechanisms.

Ultimately, to understand the function of a new gene, one should exploit all available experimental data sources (e.g., transcriptomics, proteomics, protein-protein interactions and metabolomics) [13,14] or even by the joint efforts of many scientists in a community annotation [15]. Previous work on gene function prediction has mainly been focused on higher organisms using multiple high-throughput data sources [16-18]. On the other hand, genome organizational principles that are unique for prokaryotes supply valuable additional information about gene function.

However, it is expected that the GBA method is particularly powerful for prokaryotes, due to their tight coupling of transcription and translation [19]. In addition, for many prokaryotes, the available gene expression datasets greatly outnumber other experimental data sources.

To improve the analysis of the predictions, Prosecutor provides additional information for each annotated gene, most notably in its genomic context, which is particularly useful for operons. The occurrence of adjacent divergent co-expressed genes is also highlighted since these are expected to be co-regulated [20]. Finally, putative new members of transcriptional modules are examined for the presence of the same regulatory motif that is already known for the module.

Our Prosecutor software imposes no constraints on the biological annotations used; it generates hypotheses based on large variety of annotation sources e.g., Gene Ontology, metabolic pathways, UniProt keywords, etc. This is in contrast to most other methods [11,12,16-18,21-24] which, with few exceptions [8,10], are focused on coupling genes to Gene Ontology sources only.

We discuss some of the functional assignments obtained by Prosecutor, as well as a number of mining capabilities provided by the software. We find that the increasing variety of experimental conditions used in DNA microarray experiments has greatly improved the ability to identify the function of unknown genes using GBA principles.

## Results and discussion

### Prosecutor software

Prosecutor is a standalone application developed in Java and shares its functional database structure with the FIVA software [25]. It features an iterative implementation of the GBA method which is based on iterative Group Analysis algorithm (iGA) [26]. Several characteristics of the software analysis modules are described below.

### The Iterative Guilt-By-Association (iGBA) method

**Figure 1:**
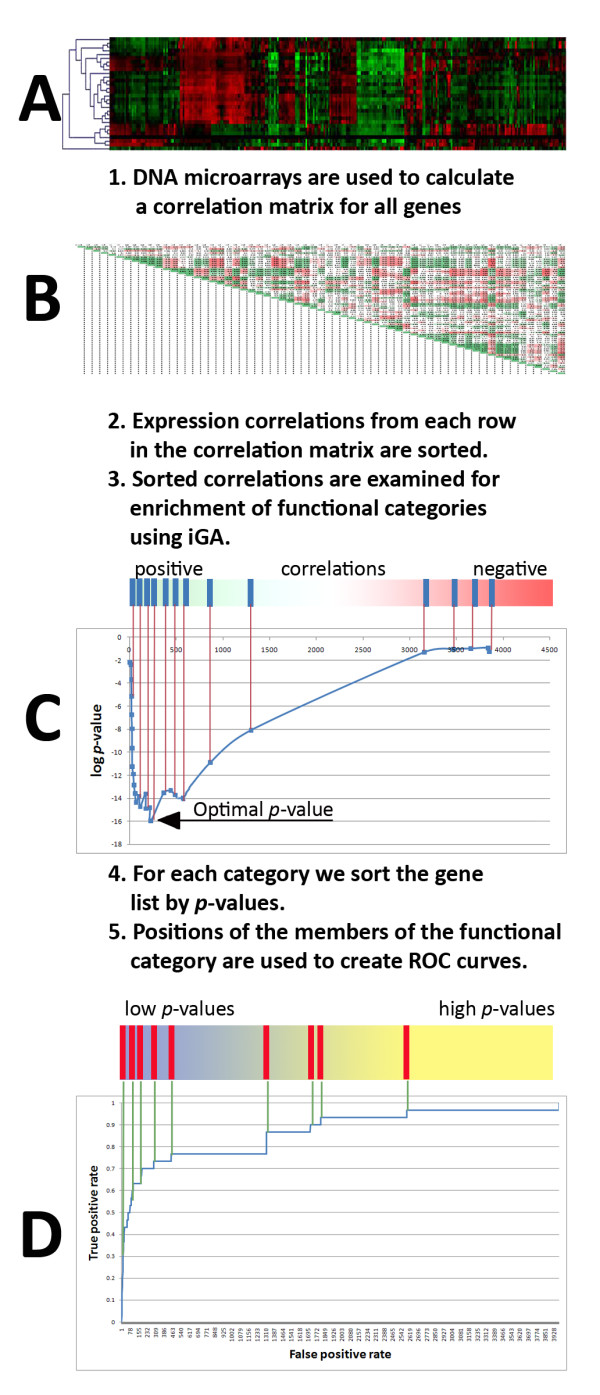
**Flowchart of Prosecutor**. Flowchart of the functional prediction process in Prosecutor. First, the expression profiles from DNA microarrays (1A) are used to create a correlation matrix (1B). For every gene, the correlations with the remaining genes are retrieved from the correlation matrix and sorted (1B2). The sorted gene list is used to perform an iterative Group Analysis for every functional category (1B3). The resulting *p*-value is indicative for the prediction of a gene as a member of a functional category (1C). At this step, the regular iGBA process ends. However, to also assess the reliability of each prediction, the following steps are added. The complete list of *p*-values for every functional category is sorted (1C4), after which the positions of the members of the functional category are determined (1C5). These positions are used to create ROC curves (1D; see Results section for more information concerning ROC curves). The corresponding Area Under the ROC Curve (AUC) is then used as a measure of expression coherence value of a functional category.

The iGBA method requires DNA microarray datasets and functional categories from annotation sources to infer putative gene functions. The rationale for our approach is the GBA principle, i.e., genes that are functionally involved in, or linked to, the same function will in general show higher expression correlations than genes that are not functionally related. The prediction algorithm of Prosecutor calculates the significance of association for all pairs of genes and functional categories. For *n *genes, expression profiles from DNA microarrays (Fig. 1A) are used to create an *n *× *n *correlation matrix M (Fig. 1B). Each row *j *of this matrix represents the (Pearson or Spearman) expression correlation between gene *g*_*j *_and all other genes. To annotate each gene *g*_*j*_, we sort all other genes by their correlation with gene *g*_*j*_, and subject the resulting sorted gene list to iGA (Fig. 1C). This results in a list of functional categories that are over-represented among the genes that are highly correlated with gene *g*_*j*_, with associated *p*-values. The iGA algorithm works iteratively and therefore does not require a fixed cutoff of the sorted correlation list, no minimum correlation has to be defined. Instead, iGA determines the appropriate cutoff that yields the lowest *p*-value for each individual analysis of a gene to a functional category. As a consequence, the function assignment by iGA is very sensitive [26] compared to methods which use a predefined correlation cut-off.

### Performance of functional categories

#### Receiver Operating Characteristic curves

The performance on well-annotated genes was assessed to evaluate the sensitivity of the iGBA method. This evaluation has to be specific for each functional category, because for some of them we expect that all members show close correlation, while others are so general that their members will not correlate and iGBA is expected to fail. The category specific evaluation of expression coherence is done as follows: Our iGBA algorithm yields a *p*-value for every pair of gene-functional category pair (Fig. 1C). This *p*-value is indicative of the confidence of the assignment of a gene to a functional category. For each category we sort the gene list by *p*-values and examine the positions of the *p*-values of its known members in this sorted list. We are then able to calculate an "expression coherence value" for each functional category by plotting the true and false positive rates on Receiver Operating Characteristic (ROC) curves (Fig. 1D) [27]. The corresponding Area Under the ROC Curve (AUC) is a quantitative measure of the expression coherence of the genes of a functional category. A functional category in which all known members show strong co-expression will have an AUC close to 1.0, whereas a randomly predicting functional category (i.e., a category that does not show coexpression of its members) would yield AUC values around 0.5. Using the AUC measure, we are now able to select the most promising functional categories for further analysis.

### Parameter free approach

Various methods have been developed that specifically employ data from microarrays studies [21-24]. Some of these methods are designed for temporal gene expression profiles [23,24] or calculate a functional enrichment for each dataset [22]. Other approaches require preprocessing of the annotation data, e.g., generating a set of validated and highly unlikely associations (see [28] for more information) used for training of the prediction model [21]. Our Prosecutor application improves on previous methods by providing a parameter free approach for the inferral of gene function. No trusted set of functional associations between proteins is required since Prosecutor treats every functional category individually, thereby circumventing preselection toward particular processes.

### Additional layers of information

The strength of Prosecutor comes also from its additional prokaryote-specific layers of information combined with a convenient visualization of the functional predictions. This prioritizing of the results allows for the rapid identification of the most promising function predictions.

#### Genomic context analysis

**Figure 2:**
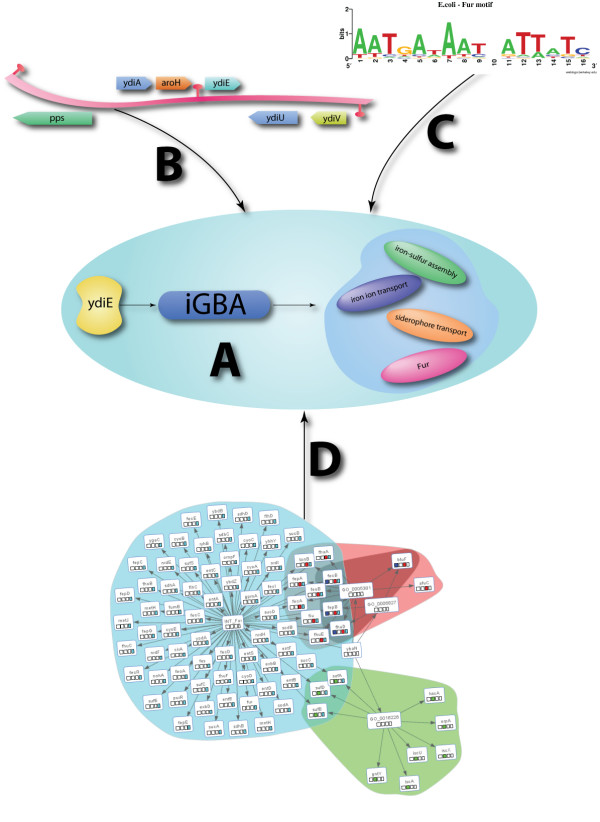
**Schematic overview of the additional information provided by Prosecutor**. Various layers of information are supplied for the iGBA results (2A) from Prosecutor. Predicted functional assignments for genes whose operon members are already linked to the predicted function are indicated in the results (2B). In addition, this protocol is also followed for divergent genes that share the same upstream region (in this example *pps *and *ydiA*). The operon information that is used for the genomic context analysis is also used to detect known regulatory sequences for transcriptional modules (2C). Lastly, graph visualization is used to visualize the gene redundancy of the different functional assignments of Prosecutor (2D). Nodes in the graph represent functional categories and genes. Arrows represent membership of gene nodes to a functional category node as well as the putative functional prediction of the studied gene. The members of individual categories are placed in colored aggregates. In addition to the aggregates, a colored square is placed in each gene member of a category. The squares are colored using the colors of their matching aggregates. Members of different categories can easily be distinguished using the colored squares. An example of a functional prediction found by Prosecutor for *ydiE *from *E. coli *is shown. The expression of this gene was correlated with members of various functional categories involved in the uptake of iron. In addition to the functional association with the transcriptional module Fur, the upstream region of *ydiE *also contains a putative Fur DNA binding site.

The function predictions generated by Prosecutor are provided for individual genes. Genes co-transcribed to a polycistronic messenger RNA are known as operons whose members typically share biological function. Predictions for genes of which other member(s) of the same operon were already linked to the predicted function are highlighted in the visualization of the results. The same procedure is applied to divergent genes which share the same upstream region (Fig. 2B). This layer of information that is based on the genomic context of genes provides additional, and in some case cases vital, information concerning putative function predictions.

#### Regulatory mechanism analysis

Transcriptional modules represent genes that are regulated by a common regulator. The regulatory mechanisms underlying the co-expression of members of a transcriptional module are used as additional evidence to prioritize the Prosecutor results. For some organisms, functional annotations based on curated knowledge of transcriptional modules are available [29,30]. Motif instances from all members of a transcriptional module are used to create a position specific scoring matrix. This matrix is used to search for additional hits in the upstream and coding regions from the first gene of the operon as well as the gene of interest (in case of residing in an operon). Using this approach, we are able to predict putative new targets for transcriptional modules that exhibit significant co-expression with known members of the transcriptional module and a putative regulatory motif in their upstream regions (Fig. 2C).

#### Graph visualization

Functional predictions are represented by Prosecutor as graphs using the Prefuse toolkit [31] to visualize the gene redundancy and overlap between the functional categories of different functional predictions. This method allows to visually determine the uniqueness of each of the function predictions. A force-directed layout from the Prefuse visualization framework is used to position the different nodes (genes) in the network (Fig. 2D).

### Performance compared to random microarray data

**Figure 3:**
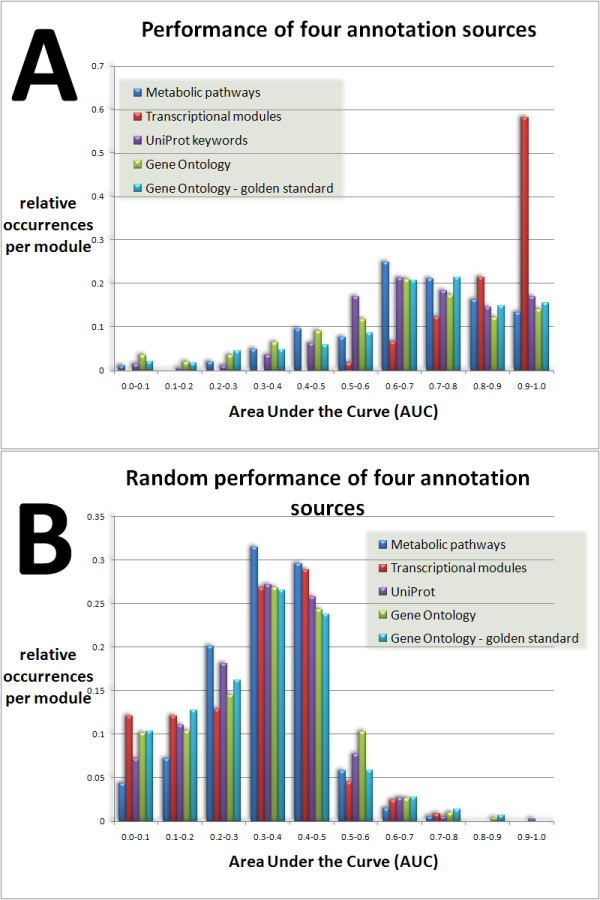
**Prediction ability of four annotation sources**. Histograms of ROC areas (Area Under the Curve) for four annotation sources for *E. coli *based on 305 microarrays (3A) compared to randomized results (3B). The real data reveal a large amount of categories with AUC values larger than 0.8, which are almost absent in randomized results. These categories are the most promising candidates for which the iGBA approach will enable confident gene assignments functional predictions. Analysis of the AUC distribution across the annotation sources shows that the "transcription module" annotation source is the most informative, i.e., contains the largest amount of categories exceeding an AUC value of 0.9 (3A). This is intuitively very convincing as shared transcriptional regulation is the basis of coexpression. In addition to ROC areas for all GO terms, we have also analyzed the distribution of ROC areas for the GO annotation source using the "gold standard" [28]. This proposed "gold standard" (GS) consists of a specific trusted set of biological processes that maps proteins to well-defined functional classes to evaluate predictions. The authors supply a set of biological processes that is based on selection by a panel of biology experts. We have included AUC results for the GO annotation for *E. coli *using the GS. Analysis of the AUC distributions shows that the distribution of relative occurrences of the GS analysis and the analysis using a fixed member cutoff is comparable.

The performance of different annotation sources (e.g., Gene Ontology terms) was investigated by comparing AUC results for real and random data using a two-sample Kolmogorov-Smirnov test. This method was used to compare the distribution of AUC values of our algorithm based on 305 microarrays from *E. coli *(Fig. 3A) as compared to results for which the genes were randomized (the link between expression and annotation is expected to be lost) (Fig. 3B). The null hypothesis that the true data do not significantly deviate from the random distribution is rejected with a *p*-value of 2e-16. The real data yield significantly higher AUC values than expected by chance. This confirms that the coexpression enrichment of many functional categories is predictive of gene function. Additional analysis of the AUC distribution across the annotation sources shows that the transcription module annotation source contains a large number of high scoring functional categories (i.e., categories exceeding an AUC value of 0.9). Moreover, we found that applying a Pearson correlation measure for calculating the correlation matrix outperforms Spearman correlations, generating 16% more functional categories with an AUC value of 0.8 or higher (data not shown).

### Prosecutor test-cases

Most genome annotations deposited to GenBank are rarely if ever updated [32]. As research progresses, knowledge of many previously uncharacterized genes improves. This annotation gap enables us to analyze results obtained by Prosecutor by manual literature mining of genes for which no function was available in the original genome annotation. For this validation, only functional categories exhibiting strong predictive properties, with AUC values higher than 0.7, were taken into account.

#### First test-case: validating results of Prosecutor

The first analysis deals with results obtained from Prosecutor for all tested organisms and was based on data from dual-dye microarrays. Prosecutor predicted a large number of gene functions for previously unannotated genes which could be validated using literature information (Table 1). The complete results of this analysis is available on the supplemental website. Analysis of the results for the model organisms *E. coli *and *B. subtilis *was facilitated by the large diversity of microarray perturbation studies available. A detailed analysis for *B. subtilis *revealed that for 25% of the best 160 predictions sufficient literature data was available to positively confirm the predictions (data not shown).

**Table 1:** Confirmed results from Prosecutor

Organism	gene	functional category	rank	auc	reference
*Campylobacter jejuni*	*Cj0391c*	Pathway flagellar assembly	2	0.76	[41,42]
	*Cj1242*	GO:0003774 motor activity	3	0.75	[42]
	*Cj1316c*	GO:0019861 agellum	7	0.72	[43]

*Escherichia coli*	*yncE*	GO:0015343 siderophore-iron transmembrane transporter activity	7	0.96	[44]
	*ybiX*	UP:Enterobactin biosynthesis	1	0.99	[44]
	*cho*	GO:0009432 SOS response	17	0.92	[45]
	*ybeD*	GO:0051082 unfolded protein binding	1	0.78	[46]
	*ulaC*	GO:0019852 L-ascorbic acid metabolic process	1	0.76	[47]
	*yciW*	GO:0006534 cysteine metabolic process	10	0.82	[48]

*Bacillus subtilis*	*ypbG*	transcriptional module SigM	18	0.81	[49]
	*ykuO*	transcriptional module Fur	8	0.75	[50]
	*yviF*	GO:0006935 chemotaxis	37	0.90	[51]
	*ylxF*	Pathway Flagellar assembly	7	0.89	[52]
	*yfnE*	transcriptional module GerE	52	0.81	[53]

*Streptomyces coelicolor*	*SCBAC28G1.05*	PW:Biosynthesis of type II polyketide back- bone	1	0.99	[54]
	*SCBAC28G1.07*	PW:Biosynthesis of type II polyketide products	8	0.76	[54]

*Vibroi cholera*	*VC1688*	GO:0006826 iron ion transport	2	0.87	[55]
	*VCA0216*	GO:0019290 siderophore biosynthetic process	8	0.98	[55]
	*VC1267*	GO:0019290 siderophore biosynthetic process	6	0.98	[55]

Functional predictions identified by Prosecutor for several organisms that are confirmed using literature information. Gene: the gene for which a validated functional prediction with a functional category (column three) was found. Rank: the position of a gene in the prioritized list based on *p*-values. These *p*-values describe the functional prediction significance for every individual gene with a specific functional category. AUC: the expression coherence value for a functional category with respect to its own members. Notice the examples of genes for which a particular annotation is assigned rank 1. This means that this gene is more close associated with this functional category then any of the original known members of the category, indicating a very high confidence in the prediction.

#### Second test-case: extending transcriptional modules in *E. coli*

The second analysis dealt with the detection of putative new members of existing transcriptional modules in *E. coli *(Table 2). We used gene expression data from 305 Affymetrix genechips [33] combined with functional annotations based on curated regulatory network information from RegulonDB [30]. The results of Prosecutor were supplemented with data obtained from the position specific scoring matrices. These matrices were based on aligned motif sequences of the known DNA binding sites from the members of every transcriptional module. We found that some of the newly identified putative transcriptional module members had been confirmed in the literature, but are not yet catalogued in RegulonDB. The remainder of the putative transcriptional module members which could not be verified using literature information are marked "putative" in Table 2. Due to the exceptional predictive performance (almost 60% of the transcriptional modules shows an AUC value above 0.9) and the additional analysis of the results using known regulatory mechanisms, we were able to reliably predict a large number of putative and validated members for transcription modules.

**Table 2:** Extending transcriptional modules of E. coli

transcriptional module	gene	prosecutor rank	motif rank	motif sequence in the intergenic region of either the gene or its operon	literature reference
**ArgR **Amino acid biosynthesis: Arginine. **AUC 0.92**	*artJ*	21	8	TGCATAACATTGCG	[56]
	*aroP*	58	39	TGATTTTTAATTCA	[57]
	*artI*	131	50	TGCATAATTATTCT	[56]
	*hisL*	16	4	TGAATAAACATTCA	putative
	*pyrL*	32	61	TGACTTTTAATTCA	putative
	*metH*	36	76	TGAATTTTTATTAA	putative
	*ydcS*	43	63	TGAATAAATTTTCT	putative
	*stpA*	132	21	TGCATTTTTATTCA	putative
	*hisG*	141	8	TGAATAAACATTCA	putative
	*hisJ*	144	27	TGCATTGAAATGCA	putative
	*hisC*	145	13	TGAATAAACATTCA	putative
	*hisA*	147	14	TGAATAAACATTCA	putative
	*potF*	162	46	TGCATAAAAATTTG	putative

**CysB **Amino acid biosynthesis: Cysteine **AUC 0.91**	*sbp*	12	0	CGCAAGTTATAGCCAATCTTTTTTTATTCTT	[48,58]
	*nlpA*	36	17	CAGACTTTATATTCCACTTTTATTCCTTTTT	[48]
	*mmuP*	28	40	AACGCGGTATAACAAACCTTCTTTGGATGTT	putative

**Fur **iron regulatory gene **AUC 0.84**	*yncD*	74	32	GGGAATGGTAATCATTATT	[44]
	*ybaN*	37	5	GAAAATGATAATTGTTATG	putative
	*folE*	101	29	GGCAATTACAATAATTATC	putative

**LexA **major regulator of DNA repair **AUC 0.87**	*yebG*	0	21	CTGTATAAAATCACAG	[59,60]
	*dinI*	2	6	CTGTATAAATAACCAG	[61,62]
	*dinB*	6	51	CTGTATACTTTACCAG	[63]
	*dinD*	19	0	CTGTATATAAATACAG	[45]
	*yjiW*	39	20	CTGATGATATATACAG	[45]
	*ybfE*	120	31	CTGATTAAAAACCCAG	[45]
	*sbmC*	125	4	CTGTATATAAAAACAG	[64]

**MetJ **Amino acid biosynthesis: Methionine **AUC 0.88**	*ybdH*	10	6	AGACGTTTAGATGTCT	[65]
	*ybdL*	106	0	AGACATCTAAACGTCT	[65]
	*ycbK*	198	17	AGTCATCTTGACGTCT	[65]
	*mmuP*	14	15	GGATGTTTAGATGTCC	putative

Transcriptional module member predictions identified by Prosecutor applied to well-known transcriptional modules for *E. coli*. Gene: the gene for which a validated functional prediction with a transcriptional module (column three) was found. Rank: represents the position of a gene in the sorted list with *p*-values. These *p*-values describe the functional prediction significance for every individual gene with a specific functional category. More significant *p*-values are matched with lower ranks. AUC: describes the association efficiency for a transcriptional module with respect to its own members. In addition to the rank information provided by Prosecutor, supplemental motif information is provided. This data is obtained by applying a position specific scoring matrix (PSSM) to the upstream sequences of all genes in the genome. The PSSM is derived from aggregating all known consensus target sequences (DNA regulatory binding sites). The additional motif information allows users to concentrate on genes that exhibit coexpression with a transcriptional module as well as possessing a predicted consensus sequence. This additional evidence contributes to the confidence in assigning a gene to a particular transcriptional module. Motif rank: based on the results for a PSSM when matched to every upstream sequence in the genome. For example, based on the PSSM of the regulator LexA, the upstream region of gene *dinD *contains the best ranking motif (rank 1).

#### Third test-case: performance of annotation sources for Saccharomyces cerevisiae

**Figure 4:**
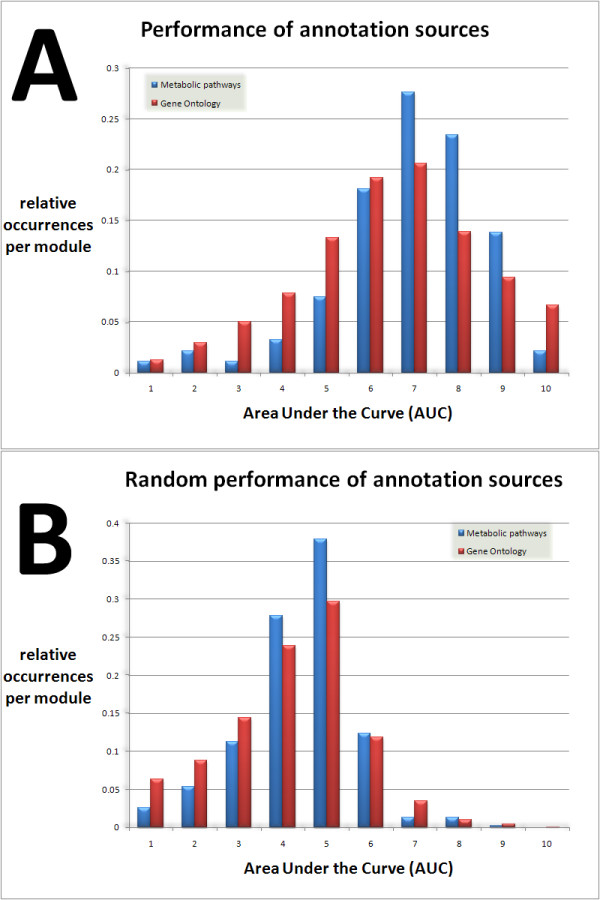
**Prediction ability of two annotation sources for yeast**. Histograms of ROC areas (Area Under the Curve) for two annotation sources (Gene Ontology and metabolic pathways) for *S. cerevisae *based on 1079 datasets from Stanford microarray database (4A) compared to randomized results (4B). The real data reveal a large number of categories with AUC values larger than 0.8, which are almost absent in randomized results. These categories are the most promising candidates for which the iGBA approach will enable confident gene assignments of functional predictions.

The genome annotation of *S. cerevisae *is available in Genbank as well as EMBL format, allowing our Prosecutor software to perform an iGBA analysis. For this third analysis we used two annotation sources (metabolic pathways and Gene Ontology). The gene expression data was obtained from the Stanford microarray database [34]. The distribution of AUC values of our algorithm (Fig. 4A) is compared to results for which the genes were randomized (Fig. 4B) The results based on the real data yield more large AUC values than expected by chance. The categories with high AUC values will presumably allow our iGBA method to assign reliable functional predictions. This demonstrates that Prosecutor, while being specifically optimized for prokaryotes, will also be a useful tool for the general biologist community.

#### Community resource

The complete results of the annotation efforts from our software for twelve organisms are available on the supplemental website [35]. On this dedicated web-site functional couplings can be mined in three ways: 1) through a list of the best functional couplings for each functional category; this allows "browsing" through the most promising associations, 2) a sorted list of functional categories and their predictive power (AUC); in case that one is interested in the genes that are associated with a specific functional category, and 3) a sorted list of genes; allows to identify to which functional categories a gene of interest is associated. All data sources used for analysis are available, allowing researchers studying any of the analyzed organisms to perform a functional analysis for their expression dataset and/or functional categories.

## Conclusion

Prosecutor uses DNA microarray data combined with functional annotations to infer putative gene functions. We show that multiple annotation sources are informative and non-redundant and allow maximizing the use of all available DNA microarray data. For *B. subtilis*, we were able to confirm 40 out of the 160 best functional predictions generated by Prosecutor, using published literature. We therefore believe that the other functional assignments based on our analysis are also likely to be informative and reliable. Combined with regulatory motif information for the species *B. subtilis *and *E. coli*, Prosecutor allows the identification of new transcriptional module members. Prosecutor can thus serve as a generic tool for a genome-wide (re)annotation of gene functions in prokaryotes. The results of such a re-annotation effort, for 11 widely studied bacterial species, is supplied as a community resource at the associated website [35].

## Methods

### Implementation & Availability

Prosecutor was programmed as a multithreaded standalone application in Java using the Eclipse framework  as a Rich Client Platform (source code is available upon request). Prosecutor runs on all Java-supporting operating systems (MS Windows, Linux and Mac OS). The Prosecutor was developed from a bacterial perspective and therefore supports the two major prokaryotic genome annotation formats (Genbank and EMBL). A simplified tabulated genome annotation format can also be used, enabling organisms for which no Genbank or EMBL file is available to be studied.

### Data sources

The basic requirements of an analysis consist of a genome annotation (i.e., Genbank or EMBL) and a collection of microarray data. Currently, six different annotation sources are implemented: (i) transcriptional modules, (ii) gene ontologies (GO) [36], (iii) metabolic pathways from the KEGG database [37] (iv) UniProt keywords [38], (v) InterPro domains [39] and (vi) user-defined categories.

### DNA microarray datasets

DNA microarray data used in this study consisted of dual dye arrays for 11 prokaryotic organisms and yeast from the KEGG expression database [40] and the Stanford microarray database [34]. For *E. coli*, an additional 305 Affymetrix expression arrays were obtained from the M3D Database [33].

### Multiple testing correction

A typical problem in genome-wide statistical analysis is the occurrence of many false positives (i.e., a functional prediction that is mistakenly found significant due to multiple testing). The incidence of false positives is roughly proportional to the number of tests performed. Since a typical search in Prosecutor may consist of thousands of tests, the chance of obtaining false positive predictions is large. We have used a strict Bonferroni multiple testing correction method to correct the raw *p*-values from the iGA results to minimize this problem.

## Authors' contributions

EJB conceived the study and programmed the software. EJB and RB devised the iGBA algorithm. KJH designed and programmed the analysis interface. EJB and SAFTH wrote the manuscript. JBTMR and OPK guided and coordinated the project. All authors read, corrected and approved the final manuscript.
